# Psychoticism in schizophrenia and sarcopenia: a case report

**DOI:** 10.1192/j.eurpsy.2026.12173

**Published:** 2026-07-31

**Authors:** M. Galetić, M. Ivandić, J. Merćep, M. Pernat, N. Rančić, T. Glavina, T. Mastelić

**Affiliations:** Department of Psychiatry, University Hospital of Split, Split, Croatia

## Abstract

**Introduction:**

Sarcopenia is rarely the subject of research in psychiatry. It is particularly poorly researched in people suffering from schizophrenia. Sarcopenia represents the loss of muscle mass and strength and thus function. It increases the risk of falls, fractures, hospitalization, disability, pneumonia, and death. It also increases the cost of treatment. People suffering from schizophrenia have an increased risk of developing sarcopenia due to a sedentary lifestyle, endocrine changes, reduced functionality, and the effects of pharmacotherapy. Also, there are common inflammatory etiological mechanisms shared by sarcopenia and schizophrenia.

**Objectives:**

To present the case of a person who developed sarcopenia due to psychotic symptoms.

**Methods:**

An examination of the medical records revealed that the patient was treated in a psychiatric clinic with psychopharmacotherapy and psychotherapy. The patient underwent standard psychiatric treatment and received pharmacotherapy, psychosocial support, and physical therapy.

**Results:**

The patient did not leave the house for two years before coming to the hospital and rarely got out of bed, and in recent months she has not gotten up at all. She states that she developed a feeling of warmth which was least in the lying position. Also, she only consumed cold food and ate less and less over time. Upon arrival at the hospital, she was malnourished and had a weak osteomuscular structure. She needed help getting up and moving. SARC-F was 8. In addition to physical therapy, the patient was given a protein-rich diet and protein supplements. She was treated with risperidone and olanzapine. During the treatment, which lasted for a month, there was a partial withdrawal of the acute psychopathological symptoms, as well as an improvement in the somatic status. SARC-F was 4 at the end of treatment.

**Conclusions:**

From the presented case it is clear how psychoticism can directly lead to sarcopenia, causing immobility and loss of appetite leading to a decrease in muscle mass, strength, and function. However, psychoticism and sarcopenia share inflammatory processes. Elevated levels of interleukin 6 were found in people with sarcopenia and people with schizophrenia. Interleukin 6 promotes catabolism and thus contributes to the development of sarcopenia. It also affects the level of serotonin in the hypothalamus, which regulates appetite and thus contributes to sarcopenia. Elevated levels of S100B protein were also found in both disorders. This is associated with general inflammation, neuroinflammation, dysfunction of astrocytes, and reduction of myogenic potential, which favors the development of sarcopenia. Certainly, additional research is needed on sarcopenia in people suffering from schizophrenia, as well as on the common pathophysiological basis.

**Disclosure of Interest:**

None Declared

